# HIF2A Variants Were Associated with Different Levels of High-Altitude Hypoxia among Native Tibetans

**DOI:** 10.1371/journal.pone.0137956

**Published:** 2015-09-14

**Authors:** Zhuoma Basang, Boyang Wang, Lei Li, La Yang, Lan Liu, Chaoying Cui, Gongga Lanzi, Nima Yuzhen, Ji Duo, Hongxiang Zheng, Yi Wang, Shuhua Xu, Li Jin, Xiaofeng Wang

**Affiliations:** 1 Medical College of Tibet University, Tibet Autonomous Region 850002, China; 2 MOE Key Laboratory of Contemporary Anthropology, School of Life Sciences, Fudan University, Shanghai 200433, China; 3 People's Hospital of Tibet Autonomous Region, Tibet Autonomous Region 850000, China; 4 Shigatse Center for Disease Control, Tibet Autonomous Region 857000, China; 5 Key Laboratory of Computational Biology, CAS-MPG Partner Institute for Computational Biology, Chinese Academy of Sciences, Shanghai 200031, China; CSIR-INSTITUTE OF GENOMICS AND INTEGRATIVE BIOLOGY, INDIA

## Abstract

Hypoxia inducible factors, including *HIF1A* and *HIF2A*, play central roles in response to high-altitude hypoxia and genetic variants of *HIF1A* or *HIF2A* were associated with high-altitude sickness or adaptation. However, it remains to determine whether they are associated with tolerance to different levels of high-altitude selection pressure among native Tibetans. We recruited 189 Tibetan subjects living at 2,700 meters (Low level of high altitude, LHA), 197 at 3,200 meters (Middle level of high altitude of high altitude, MHA), 249 at 3,700 meters (High level of high altitude, HHA) and 269 at 4,700 meters (Very high level of high altitude, VHA) and performed association analysis of twelve tSNPs (tagging SNPs) in *HIF1A* and *HIF2A* with high-altitude. We found (1) a increasing trend of *HIF2A* rs5621780-C(18.4%, 15.9%, 32.8% and 31.1%, respectively, in LHA, MHA, HHA and VHA)(*P* = 3.56E-9); (2) increasing trends of *HIF2A* rs6756667-A(68.7%, 73.4%, 79.9% and 89.6%), rs7589621- G(74.6%, 77.9%, 83.7%, and 92.1%) and rs1868092-A(64.1%, 67.3%, 75.1% and 84.4%) (*P* = 3.56E-9, 4.68E-16, 1.17E-13 and 7.09E-14, respectively); (3) a increasing trend of haplotype AG (68.7%, 73.1%, 79.9% and 89.6%) (*P* = 2.22E-7) which was constructed by rs6756667 and rs7589621; (4) a strong linear correlation between major alleles of rs6756667-A (*R* ^2^ = 0.997, *P* = 0.002), rs7589621-G (*R* ^2^ = 0.994, *P* = 0.003), rs1868092-A (*R* ^2^ = 0.985, *P* = 0.008) and altitude by linear correlation test. The associations between *HIF2A* variants and different level of high altitude support that extremely high-altitude hypoxia challenge imposes selective effects on *HIF2A* variants among native Tibetans.

## Introduction

Hypobaric hypoxia is a major geographic feature of high-altitude regions [1]. In high-altitude environment, the decreased oxygen availability exerts a harsh survival challenge for human beings [2]. This introduces the concept of high-altitude adaptation of irreversible long-term physical responses to high-altitude environment associated with heritable behavior and genetic changes. Tibetan is one of the major high-altitude dwellers. Since modern Tibetans immigrated to the Qinghai-Tibet Plateau as early as Neolithic age [3, 4], long-term persistent and ongoing selection has altered their genetic constitutions [5], which led to their phenotypic adaptation to high-altitude hypoxia environment [6–9]. Unlike some other high-altitude populations [10], Tibetans are less influenced by migrants and exotic cultures [11, 12]. They also have a relatively uniform lifestyle and occupation [12]. Tibetan is an ideal population to understand the biological adaptation of human beings to high-altitude environment.

Previous studies had shown that highlanders exhibited improved survival to high altitude compared with lowlanders [13, 14]. Genetic evidences had been discovered to contribute to the tolerance [15–17]. More recently, genome-wide (GW) studies, including ours, identified strong signals of selective sweep in two hypoxia-related genes, *HIF2A* (also known as *EPAS1*) and *EGLN1* [18–25]. Compared with the non-Tibetan lowlanders, Tibetans showed highly-differentiated allelic and haplotypic signals in *HIF2A* and *EGLN1*, suggesting that during the long-term occupation of high-altitude areas, the functional sequence variations for acquiring biological adaptation to high-altitude hypoxia have been enriched in Tibetan populations [5]. In addition, a higher frequency of endothelial nitric oxide synthase (*ENOS*) gene homozygotes (GG and BB) was found associated with a higher altitude and elevated level of plasma nitric oxide [26]. However, the relationships between genetic variants and different levels of high altitude among the same ethnic group were poorly understood. Moore L.G. et al. [27] compared the frequency of myoglobin (*MB*) gene A79G and T109C polymorphisms among Tibetans living at altitudes of 3,000, 3,700 and 4,500 meters and to lowlanders. They found higher frequency of the *MB* 79A allele in highland Tibetans than in lowlanders, while unchanged with increasing altitude from 3,000–4,500 meters among native Tibetans. Moore’s work inspired us to continue searching for genetic variants in other hypoxia-related genes that were involved in the processes of adaptations in Tibetans living in altitudes ranging from 2,700 meters to 4,700 meters.

Hypoxia inducible factors (HIFs) are transcription factors that directly or indirectly regulate hundreds of genes involving in angiogenesis, cell growth, apoptosis, energy metabolism and vasomotor regulation [28, 29]. HIFs comprise α subunit (HIF1A, HIF2A or HIF3A) and a constitutively expressed β subunit [30, 31]. Among them, HIF1A and HIF2A play central roles in response to hypoxia [32–34]. Genetic variants of *HIF1A* or *HIF2A* were found associated with high-altitude adaptation [24, 32], high-altitude sickness [35, 36] or tumor hypoxia [37]. Considering the roles of *HIF1A* and *HIF2A* variants in high-altitude physiological and pathological process, here we aim to test the hypothesis that specific polymorphisms of *HIF1A* or *HIF2A* genes might be associated with either tolerance to different levels of high-altitude selection pressure or physiological traits among native Tibetans.

## Methods

### Study subjects

The study included 904 healthy native Tibetans with DNA sample and demographic data. Among them, 189 subjects were recruited from Bomi County (95°75E/ 29°92´N, 2,700 meters high), 197 from Qamdo County (97°17´E/ 31°14´N, 3,200 meters high), 249 from Lhasa City (91°11´E/ 29°97´N, 3,700 meters high), and 269 from Amdo County (91°68´E/ 32°29´N, 4,700 meters high). The subjects were classified into four groups according to their living altitude: low level of high altitude group (group LHA) for Bomi subjects, middle level of high altitude group (group MHA) for Qamdo subjects, and high level of high altitude group (group HHA) for Lhasa subjects and very high level of high altitude group (group VHA) for Amdo subjects. About 45% of the subjects were male. Their age varied from 14 to 25 (Table 1). All subjects were fully ethnically Tibetan for at least three preceding generations. None of the subjects had known low-altitude progenitors.

**Table 1:** Clinical characteristics of the studied groups.

	Bomi	Qamdo	Lhasa	Amdo	Total
**N**	189	197	249	269	904
**Female (%)**	54.5	55.3	75.9	33.8	54.4
**Age (years)**	18.7±1.4^**a**^	19.9±2.6	21.1±1.9	18.2±1.3	19.5±2.1
**BMI (kg/m** ^**2**^ **)**	20.1±1.9	21.5±2.1	20.3±2.1	19.6±1.9	20.3±2.1
**RBC(×10** ^**12**^ **/L)**	5.00±0.60	5.10±0.62	4.96±0.57	5.82±0.74	5.25±0.74
**HB(g/L)**	140.84±20.92	148.08±20.90	144.17±20.60	169.28±22.18	151.76±24.15
**HCT(%)**	42.24±5.19	43.44±5.42	43.27±5.32	48.80±6.05	44.73±6.14
**LVEF(%)**	77.25±2.92	75.09±3.58	75.69±5.26	72.40±3.67	61.00±4.38

Abbreviations: BMI, body mass index; RBC, red blood cell count, RBC; HB, hemoglobin; HCT, hematocrit; LVEF, left ventricular ejection fraction.

^**a**^ Data are means ± SD.

### Ethics statements

Written informed consent was obtained from each participant and their guardians of 18 years old. All protocols were approved by the Human Ethics Committee of School of Life Sciences of Fudan University.

### Phenotypic measurements

All subjects were examined by field workers expertizing on high-altitude adaptation research. Body weight (kg) and height (m) were measured to calculate body mass index (BMI, kg/ m^2^, the body weight in kilograms divided by the square of height in meters). Blood specimens were drawn after overnight fasting for complete blood count measurement by SYSME pocH-100i. The blood count parameters included red blood cell count (RBC, ×10^12^/L), hemoglobin (HB, g/L) and hematocrit (HCT, %). Left ventricular ejection fraction (LVEF, %) was measured by Acuson CA94043 (USA). The detailed characteristics were shown in Table 1.

### Selection of tagging SNPs

Haplotype-tagging SNPs (tSNPs) of *HIF1A* and *HIF2A* were selected using the Chinese Han sample in Bejing, China, available at public data released on the International HapMap Data Phase III/ Rel #3, May 10, on NCBI B36 assembly, dbSNP b126 (http://hapmap.ncbi.nlm.nih.gov/cgi-perl/gbrowse/hapmap3r3_B36/). To identify common haplotype tSNPs, eligible SNPs were entered into the tagger program that had been implemented in Haploview version 4.2 [38]. We defined common variants as those with a heterozygosity of >5% and set a threshold of 0.8 for the linkage disequilibrium (LD) measure *r* ^*2*^). Finally, seven tSNPs (rs2301104, rs12434438, rs966824, rs2301112, rs2301113, rs11549465 and rs11549467) in *HIF1A* capturing 20 SNPs and five tSNPs (rs56721780, rs6756667, rs7589621, rs59901247 and rs1868092) in *HIF2A* capturing 96 SNPs were selected. The chromosome position, gene position, potential function significance and major/ minor allele of the selected tSNPs were summarized in Table 2.

**Table 2:** Characteristics of the studied tSNPs ^a^.

Gene	dbSNP ID	Chr	Position	Allele	Category	Function	Call rate
***HIF1A***	rs2301104	14	62165028	G/C	Intron 1	/	99.78%
	rs12434438	14	62197298	A/G	Intron 6	/	99.66%
	rs966824	14	62200518	C/T	Intron 7	/	99.78%
	rs2301112	14	62206173	A/C	Intron 10	/	99.10%
	rs2301113	14	62206548	A/C	Intron 10	/	99.78%
	rs11549465	14	62207557	C/T	Exon 12	P582S	99.89%
	rs11549467	14	62207575	G/A	Exon 12	A588T	99.78%
***HIF2A***	rs56721780	2	46523655	G/C	5’-Up	/	98.67%
	rs6756667	2	46579409	A/G	Intron 2	/	99.67%
	rs7589621	2	46582382	G/A	Intron 2	/	99.45%
	rs59901247	2	46609572	A/C	Exon 15	T766P	99.89%
	rs1868092	2	46614202	A/G	3’-Down	/	99.23%

Abbreviations: Chr: Chromosome.

^**a**^ From dbSNP (release 137).

### Genotyping

Laboratory personnel were blinded to clinical status. Blood of the subjects was taken into EDTA-containing receptacles and genomic DNA was extracted using a standard phenol-chloroform method. Genotyping of the selected 12 tSNPs was conducted using Taqman assays (Applied Biosystems, Foster City). Sample DNA (10 ng) was amplified by PCR following the recommendations of the manufacturer. Fluorescence was detected using an ABI 7900HT and the alleles were scored using Sequence Detection Software (Applied Biosystems, Foster City). The concordance rate of duplicate samples was above 99%. Sequencing was implemented to test the validity of genotyping.

### Statistical analysis

Descriptive statistics for continuous variables were summarized as mean ± standard deviation. The deviation from Hardy-Weinberg expectation was tested by a chi-square statistic on SPSS software version 19.0. Genotype and allele frequencies were established by genotype/allele counting. Linear-by-linear association was evaluated by two-way contingency tables using SPSS 19.0 or, if an expected value was less than five, by Fisher’s exact test (by R software with the package “stats”). Physiological traits were analyzed by one-way ANOVA (analysis of variance) and GLM (general liner model), in which phenotype acted as the corresponding variables, and genotype as the grouping variable. To construct related haplotype, genotype data were used to estimate inter-marker LD, measure pair-wise *D’* and *r* ^2^ and define LD blocks. Haplotypes were inferred using PHASE [39] software. Haplotype association tests were estimated using the Haploview version 4.2 [38]. Haplotypes within each LD block were tested for association with altitude with 10,000 times of permutation. The traditional Bonferroni correction was used to exclude false positive results due to multiple testing. Linear correlations of allele frequencies of genetic variants with altitude were conducted by SPSS 19.0.

## Results

### Preliminary result

The call rates of the 12 genotyped tSNPs were listed in Table 2. All tSNPs were consistent with Hardy-Weinberg expectations. Genotypes of 1% of the samples were confirmed by sequencing and were found to be 100% concordant. The minor allele frequencies (MAF) of the genotyped tSNPs ranged from 0.5% to 39.0%. The genotype and allele frequencies of the 12 tSNPs were listed in Tables 3 and 4. Since minor homozygotes rates of rs2301104, rs2301112, rs11549465, rs11549467 and rs59901247 were only 0–0.7%, the associations of them with altitudes were conducted by combining rare homozygotes with heterozygote of the tSNPs.

**Table 3:** Association analysis between *HIF1A* tSNPs and levels of high altitude among native Tibetans.

SNP	Genotype/Allele	Level of high altitude				*P*-value^b^		
		Bomi	Qamdo	Lhasa	Amdo	Additive	Dominant	Allelic
**rs2301104**	GG	176(94.6%)	182(96.3%)	236(95.5%)	262(98.1%)	0.078^**a**^	0.078	0.080
	CG	10(5.4%)	7(3.7%)	11(4.5%)	5(1.9%)			
	CC	0(0.0%)	0(0.0%)	0(0.0%)	0(0.0%)			
	C	10(2.7%)	7(1.9%)	11(2.3%)	5(0.9%)			
**rs12434438**	AA	88(47.6%)	98(51.9%)	129(52.4%)	147(54.8%)	**0.042**	**0.141**	**0.038**
	AG	77(41.6%)	71(37.6%)	94(38.2%)	106(39.6%)			
	GG	20(10.8%)	20(10.6%)	23(9.3%)	15(5.6%)			
	G	117(31.6%)	111(36.2%)	140(35.2%)	136(25.4%)			
**rs966824**	CC	98(53.0%)	103(54.5%)	142(57.7%)	155(57.8%)	0.107	0.249	0.117
	CT	76(41.1%)	74(39.2%)	91(36.8%)	106(39.6%)			
	TT	11(5.9%)	12(6.3%)	14(5.7%)	7(2.6%)			
	T	98(26.5%)	98(32.2%)	119(29.5%)	120(22.4%)			
**rs2301112**	AA	174(95.1%)	175(93.6%)	235(95.5%)	254(95.1%)	0.739^**a**^	0.739	0.742
	AC	9(4.9%)	12(6.4%)	11(4.5%)	13(4.9%)			
	CC	0(0.0%)	0(0.0%)	0(0.0%)	0(0.0%)			
	C	9(2.5%)	12(3.3%)	11(2.3%)	13(2.4%)			
**rs2301113**	AA	62(33.3%)	62(32.8%)	96(39.0%)	115(42.9%)	**0.032**	**0.014**	**0.037**
	AC	103(55.4%)	97(51.3%)	112(45.5%)	127(47.4%)			
	CC	21(11.3%)	30(15.9%)	38(15.4%)	26(9.7%)			
	C	145(39.0%)	157(55.9%)	188(49.5%)	179(33.4%)			
**rs11549465**	CC	170(91.4%)	175(92.6%)	234(94.4%)	251(94.0%)	0.229^**a**^	0.222	0.222
	CT	15(8.1%)	14(7.4%)	14(5.6%)	15(5.6%)			
	TT	1(0.5%)	0(0.0%)	0(0.0%)	1(0.4%)			
	T	17(4.6%)	14(3.8%)	14(2.9%)	17(3.2%)			
**rs11549467**	GG	184(98.9%)	178(94.7%)	232(93.9%)	256(95.5%)	0.112^**a**^	0.112	0.116
	GA	2(1.1%)	10(5.3%)	15(6.1%)	12(4.5%)			
	AA	0(0.0%)	0(0.0%)	0(0.0%)	0(0.0%)			
	A	2(0.5%)	10(2.7%)	15(3.1%)	12(2.2%)			

Abbreviations: Additive, additive model; Dominant, dominant model.

*P*-values except the noted ones are calculated from χ^2^ test.

^**a**^ *P*-values are calculated from Fisher exact test.

^**b**^ Bold type denotes *P*<0.05.

**Table 4:** Association analysis between *HIF2A* tSNPs and levels of high altitude among native Tibetans.

SNP	Genotype/Allele	Level of high altitude				*P*-value^b^		
		Bomi	Qamdo	Lhasa	Amdo	Additive	Dominant	Allelic
**rs56721780**	GG	124(66.0%)	132(68.8%)	110(44.5%)	126(47.5%)	**3.06E-9** ^**a**^	**1.53E-7**	**3.56E-9**
	CG	59(31.4%)	59(30.7%)	112(45.3%)	113(42.6%)			
	CC	5(2.7%)	1(0.5%)	25(10.1%)	26(9.8%)			
	C	69(18.4%)	61(15.9%)	162(32.8%)	165(31.1%)			
**rs6756667**	AA	95(50.8%)	105(53.3%)	156(62.7%)	214(79.9%)	**2.29E-15** ^**a**^	**5.81E-12**	**4.68E-16**
	AG	67(35.8%)	79(40.1%)	86(34.5%)	52(19.4%)			
	GG	25(13.4%)	13(6.6%)	7(2.8%)	2(0.7%)			
	A	257(68.7%)	289(73.4%)	398(79.9%)	480(89.6%)			
**rs7589621**	GG	109(57.7%)	119(60.4%)	172(69.4%)	224(84.5%)	**3.41E-13** ^**a**^	**2.96E-11**	**1.17E-13**
	AG	64(33.9%)	69(35.0%)	71(28.6%)	40(15.1%)			
	AA	16(8.5%)	9(4.6%)	5(2.0%)	1(0.4%)			
	G	282(74.6%)	307(77.9%)	415(83.7%)	488(92.1%)			
**rs59901247**	AA	161(85.2%)	166(84.3%)	227(91.5%)	243(90.3%)	0.032^a^	0.020	0.033
	AC	27(14.3%)	31(15.7%)	21(8.5%)	24(8.9%)			
	CC	1(0.5%)	0(0.0%)	0(0.0%)	2(0.7%)			
	A	349(92.3%)	363(92.1%)	475(95.8%)	510(94.8%)			
**rs1868092**	AA	81(43.1%)	86(43.9%)	135(54.7%)	190(71.4%)	**1.12E-13**	**3.54E-11**	**7.09E-14**
	AG	79(42.0%)	92(46.9%)	101(40.9%)	69(25.9%)			
	GG	28(14.9%)	18(9.2%)	11(4.5%)	7(2.6%)			
	A	241(64.1%)	264(67.3%)	371(75.1%)	449(84.4%)			

Abbreviations: Additive, additive model; Dominant, dominant model.

*P*-values except the noted ones are calculated from χ^2^ test.

^**a**^ *P*-values are calculated from Fisher exact test.

^**b**^ Bold type denotes *P*<0.05.

#### Association between HIF2A tSNPs and levels of high altitude

Both genotype and allele (Table 4) patterns distributed differently in LHA, MHA, HHA and VHA subjects. We detected four *HIF2A* tSNPs (rs56721780, rs6756667, rs7589621 and rs1868092) significant associated with level of high altitude among native Tibetans. The major allele of rs6756667, rs7589621 and rs1868092 was associated with a higher level of altitude. Moreover, the significance remained after applying a Bonferroni correction for multiple testing and gender-wise comparison (Table A and B in S1 File).

For rs5621780, linear-by-linear association test revealed a significant increasing trend of minor C allele frequency from LHA (18.4%), MHA (15.9%) and HHA (32.8%) to VHA (31.1%) (*P* = 3.56E-9; adjusted *P* = 1.07E-8). For rs6756667, linear-by-linear association test revealed a significant increasing trend of major A allele frequency from LHA (68.7%), MHA (73.4%) and HHA (79.9%) to VHA (89.6%) (*P* = 4.68E-16; adjusted *P* = 5.62E-15). In addition, the frequencies of major genotype AA (50.8%, 53.3%, 62.7% and 79.9%) also linearly increased in LHA, MHA, HHA and VHA.

For rs7589621, linear-by-linear association test revealed a significant increasing trend of major G allele frequency from LHA (74.6%), MHA (77.9%) and HHA (83.7%) to VHA (92.1%) (*P* = 1.17E-13; adjusted *P* = 4.68E-12). In addition, the frequencies of major genotype GG (57.7%, 60.4%, 69.4% and 84.5%) also linearly increased in LHA, MHA, HHA and VHA. For rs1868092, linear-by-linear association test revealed a significant increasing trend of major A allele frequency from LHA (64.1%), MHA (67.3%) and HHA (75.1%) to VHA (84.4%) (*P* = 7.09E-14; adjusted *P* = 4.25E-13). In addition, the frequencies of major genotype AA (43.1%, 43.9%, 54.7% and 71.4%) also linearly increased in LHA, MHA, HHA and VHA.

**Fig 1:**
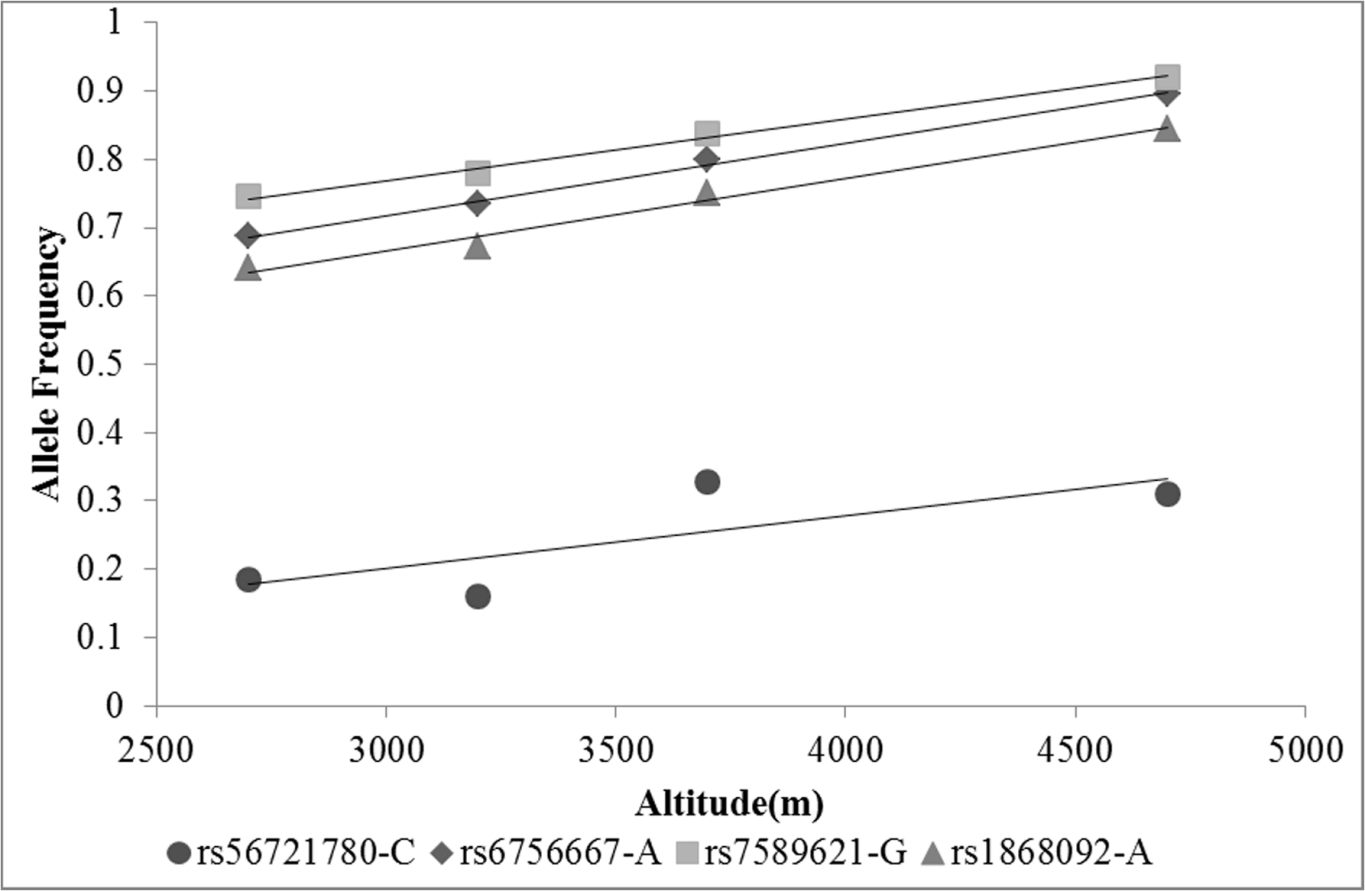
Correlation of allele frequencies of *HIF2A* variants in Tibetans and level of high altitude. solid circle, rs56721780-C; solid diamond, rs6756667-A; solid square, rs7589621-G; solid triangle, rs1868092-A

Linear correlation analysis revealed that the major A allele of rs6756667 (*R* ^2^ = 0.997, *P* = 0.002), major G allele of rs7589621 (*R* ^2^ = 0.994, *P* = 0.003) and major A allele of rs1868092 (*R* ^2^ = 0.985, *P* = 0.008) were highly correlated with level of high altitude. The minor C allele of rs5621780 (*R* ^2^ = 0.593, *P* = 0.230) was in moderate correlation with level of high altitude. (Fig 1)

### LD analysis of the altitude-associated HIF2A tSNPs

Haplotype analysis revealed that rs6756667 was in moderate LD with rs7589621 (pair-wise *r* ^2^ = 0.76) which are 2,973 bp apart (data not shown). A common haplotype AG defined by rs6756667 and rs758962 increased linearly in LHA, MHA, HHA and VHA with frequencies of 68.7%, 73.1%, 79.9% and 89.6%, respectively (*P* = 2.22E-7). The pair-wise LDs constructed by other tSNPs were weak among all altitude groups.

#### Association between HIF1A tSNPs and levels of high altitude

The *HIF1A* rs12434438 and rs2301113 showed marginal associations with level of high altitude at the significance level of α = 0.05 (Table 3). However, the associations disappeared when adjusting for Bonferroni correction. In addition, no associations of other *HIF1A* tSNPs with altitude were found.

### Association between HIF tSNPs and physiological traits

The CG genotype of *HIF2A* rs1868092 was associated with a higher level of LVEF (76.80%), compared with GG genotype (74.85%) (p = 0.011). The significance remained after controlling for covariates of age, gender and altitude (p = 0.045). In addition, no significant association was found between other tSNPs and RBC, HB, and HCT.

## Discussion

### Characteristics of the present study

The Qinghai-Tibet Plateau is a territory with a sparse population, especially at very high altitudes. According to the Fourth National Census of China conducted in 1990, about 6.19% of Tibetans (284,558 out of 4,594,188) lived in an altitude exceeding 4,500 meters and 1.02% above 5,000 meters in the 240 square-kilometers Qinghai-Tibet Plateau [40]. The VHA subjects recruited in the present study live at 4,700 meters height, an altitude few high altitude researchers touched upon due to the harshness of the environment and inconvenience of transportations.

Up to now, most high-altitude adaptation studies [18–24] focused on highlanders at lower than 3,500 meters, comparing the physiological traits or genetic differences of highlanders with those of sea level dwellers. These comparisons had discovered the selective signals towards hypoxic stress. However, our aim was to elucidate whether highlanders were further genetically selected among different levels of high altitude, even at an extreme altitude of 4,700 meters, finding that extremely high-altitude hypoxia challenge imposed selective effects on polymorphisms and haplotypes of *HIF2A* among native Tibetans.

Another characteristic of this study was the small probability of genetic admixture or genetic drift, both inter-group (among four different high-altitude groups) and inter-population (between the studied Tibetan natives and nearby lowlanders), which decreased the genetic heterogeneity of the studied population and the possibility of sampling error. No report of outside contact or large-scale immigration was found in the studied regions in recent years [41]. In addition, the harsh environment of the Qinghai-Tibet Plateau increased the difficulties for Tibetans to move to higher or lower altitudes [42]. In fact, more than three preceding generations of the studied subjects were born and permanently lived at the same altitude.

### Association between HIF2A variants and levels of high altitude

In a previous study [5], we proposed that both haplotype-based and allelic frequency-based methods should be performed in detecting altitude selection signals in case natural selection initiated long time ago (i.e., for Tibetans) and particularly in genomic regions with recombination hotspots. In this study, we found that not only a haplotype (AG haplotype constructed by rs6756667 and rs7589621 that locate 2,973 bp apart), but also three allelic frequencies changed linearly with altitude.

Since most of these alleles were Tibetan-dominated (i.e., rs6756667-A or rs1868092-A were more than 84% for Tibetans living in a height of 4,700 meters while less than 14% for lowland Japanese and Chinese Han), they may represent the alleles that increase fitness to high-altitude hypoxia (Table 5). Rs1868092 is located in 3’-UTR of the *HIF2A* gene and 31,820 bp from rs7589621 with weak pair-wise LD. The rare allele of rs1868092 that linearly increased with the increase of altitude (LHA, MHA, HHA, and VHA), may represent a biological functional variant somewhere that increased the function of *HIF2A*, and hence increased adaptive ability to high-altitude hypoxia. Rs6756667-rs7589621 and rs1868092 may represent two haplotype blocks of selective sweeps under high-altitude hypoxia conditions. However, the pleiotropic effects of multiple genetic variants, either in the same gene or other genes, may counteract the increase of the fitness to high-altitude hypoxia. For example, the major G allele frequency of *HIF2A* rs56721780, another SNP in the 5’-upstream of *HIF2A* that is not in LD with both rs6756667-rs7589621 haplotype block or rs1868092, decreased with altitude among our native Tibetan samples. The variants rs56721780 [although only 18.4% in LHA, 15.9% in MHA, 32.8% in HHA and 31.1% in VHA, contrasting sharply with the extreme low frequencies of the low altitude Chinese Han (1%)] (Table 5) may represent a haplotype that is suffering ongoing selection by very high altitude of the Tibetans in this region, which is yet to be studied. To be mentioned, genetic variants in the 5’-upstream of specific genes may be particularly important, in that it may be located in a transcription promoter [43] or enhancer [44]. Further studies need to be carried out to address this issue.

**Table 5:** Allele Frequencies of the altitude-associated *HIF2A* tSNPs between populations.

SNP	Non-Tibetans		Tibetan populations					
	JPT^a^	CHB^a^	2700 m	3200 m	3700 m	4700 m	TBT^b^	TBQ^c^
**rs56721780(C)**	2.2%	1.0%	18.4%	15.9%	32.8%	31.1%	/^**d**^	/^**d**^
**rs6756667(A)**	8.4%	13.9%	68.7%	73.4%	79.9%	89.6%	73.3%	72.6%
**rs7589621(G)**	23.0%	27.3%	74.6%	77.9%	83.7%	92.1%	75.0%	77.4%
**rs1868092(A)**	9.6%	8.2%	64.1%	67.3%	75.1%	84.4%	75.0%	67.7%

Abbreviations: TBT, Tibetan in Tibet; TBQ, Tibetan in Qinghai; CHB, Chinese Han in Beijing. Japanese in Tokyo, Japan.

^**a**^ From 1000 GENOMES, phase 1.

^**b**^ From Xu *et al*.2011.

^**c**^ From Simonson *et al*.2010.

^**d**^ No data.

### Consistent and novel findings compared with the genome-wide studies

The associations between *HIF2A* variants and high-altitude hypoxia stress we detected in Tibetans were consistent with the previous GW discoveries, although theirs focused on the comparison between high-altitude Tibetans and other sea level populations (i.e., Chinese Han and Japanese) while ours on Tibetan ethnic groups in different levels of high altitude. In the past three years, seven GW studies of high-altitude adaptation in Tibetan populations identified positive selection signals in genetic variants of *HIF2A* (*EPAS1*) gene [18–24], indicating that nature selection at the *HIF2A* locus occurred frequently throughout the Qinghai-Tibetan Plateau and therefore were liable to detect [45, 46]. Table 5 showed frequency differences of selected tSNPs among populations living in different altitude.

For rs6756667, rs7589621 and rs1868092, dominant alleles of Tibetan populations in the present study and studies of Xu’s and Simonson’s ranged from 64.1% to 92.1%, which sharply decreased to 8.2%-27.3% in lowland Japanese and Chinese Han. For rs56721780, minor C allele of Tibetan populations in our study ranged from 18.4% to 31.1%, which also decreased in lowland Japanese (2.2%) and Chinese Han (1%). Therefore, rarely high divergences of variants represent different haplotype blocks of *HIF2A* were observed between Tibetans and non-Tibetan lowlanders, albeit in different direction. In addition, different haplotype blocks may be further moderately selected within different levels of high altitude. According to previous studies, the high altitude selection of *HIF2A* is antique, long-term persistent and ongoing. Since the selection pressure of hypoxia in high-altitude area has not relaxed, does this imply that these variants will eventually be fixed?

### Potential mechanisms of further genetic selection of HIF2A, instead of HIF1A

Contrary to *HIF2A*, *HIF1A* variants revealed no significant association with level of high altitude among native Tibetans. It was reported that HIF1A was an acute hypoxia responder, while HIF2A responded more effectively to prolonged hypoxia [47]. Therefore, the ubiquitously-expressed, conservatively-constituted and acutely-responded *HIF1A* is less likely to be selected among native Tibetans living in different level of high altitude for generations. Unlike HIF2A, HIF1A is highly conserved [48, 49] and often recognized as the “master regulator” of cellular and systemic oxygen homeostasis [28] as it induces over 70 genes that respond to hypoxia [50–52]. In addition, all known extant metazoan species have *HIF1A*, while only vertebrates have *HIF2A* [36]. HIF1A is expressed ubiquitously, whereas HIF2A expression is limited to endothelium, kidney, lung, heart, and small intestine [53].

### Limitations, conclusions and prospective

The limitations of this study should be noted. We only genotyped 12 tSNPs of the selected genes, instead of sequencing all of the genetic variants in our subjects and other lowlanders (i.e., Chinese Han). Therefore, we cannot construct haplotype network of the suggested *HIF2A* gene to reveal the tolerances of the whole gene to different levels of high-altitude selection pressure among native Tibetans and compared with those of the sea level populations. In addition, we did not detect significant association between genetic variants and physiological traits. A possible explanation was that the sample size of this study was not large, which decreased the statistical power. However, our sample represents a relatively large proportion of population in a relatively minor homogeneous ethnicity, especially for the extremely high-altitude Amdo (4,700m) population.

In conclusion, we discovered that extremely high-altitude hypoxia challenges (different levels of high altitude above 2,500 meters, especially 4,700 meters) imposed selective effects on *HIF2A* variants among native Tibetans. Since this study was exploratory, the observations need to be replicated in other highland populations.

## Supporting Information

S1 File Association analysis between *HIF2A* tSNPs and levels of high altitude among native Tibetans by age. Association analysis between *HIF2A* tSNPs and levels of high altitude among native male Tibetans (**Table A**). Association analysis between *HIF2A* tSNPs and levels of high altitude among native female Tibetans (**Table B**).
(DOC)
