# Supplementary material for: HIF2A Variants Were Associated with Different Levels of High-Altitude Hypoxia among Native Tibetans
Source: PLoS One. 2015 Sep 14;10(9):e0137956. doi: 10.1371/journal.pone.0137956 (PMC4569405; doi:10.1371/journal.pone.0137956)
Supplement: S1 File — Association analysis between HIF2A tSNPs and levels of high altitude among native male Tibetans (Table A). Association analysis between HIF2A tSNPs and levels of high altitude among native female Tibetans (Table B). (DOC) [file pone.0137956.s001.doc]

**Table A. in S1 File Association analysis between *HIF2A* tSNPs and levels of high altitude among native male Tibet**ans.

| **SNP** | **Genotype** |  | **Level of high altitude** | | | |  | ***P*-value^b^** | | |
| --- | --- | --- | --- | --- | --- | --- | --- | --- | --- | --- |
|  | **/Allele** |  | **Bomi** | **Qamdo** | **Lhasa** | **Amdo** |  | **Additive** | **Dominant** | **Allelic** |
| **rs56721780** | GG |  | 52(60.5%) | 60(69.0%) | 25(41.7%) | 86(49.1%) |  | **4.40E-4^a^** | **0.008** | **4.86E-4** |
|  | CG |  | 33(38.4%) | 27(31.0%) | 27(45.0%) | 72(41.1%) |  |  |  |  |
|  | CC |  | 1(1.2%) | 0(0.0%) | 8(13.3%) | 17(9.7%) |  |  |  |  |
|  | C |  | 35(20.3%) | 27(15.5%) | 43(35.8%) | 106(30.3%) |  |  |  |  |
| **rs6756667** | AA |  | 44(57.0%) | 45(59.1%) | 40(75.0%) | 141(84.7%) |  | **1.90E-9^a^** | **1.60E-7** | **4.99E-10** |
|  | AG |  | 30(36.0%) | 34(34.1%) | 20(25.0%) | 35(15.3%) |  |  |  |  |
|  | GG |  | 10(7.0%) | 9(6.8%) | 0(0.0%) | 1(0.0%) |  |  |  |  |
|  | A |  | 118(70.2%) | 124(70.5%) | 100(83.3%) | 317(89.5%) |  |  |  |  |
| **rs7589621** | GG |  | 49(57.0%) | 52(59.1%) | 45(75.0%) | 149(84.7%) |  | **3.55E-9^a^** | **5.42E-8** | **1.68E-9** |
|  | AG |  | 31(36.0%) | 30(34.1%) | 15(25.0%) | 27(15.3%) |  |  |  |  |
|  | AA |  | 6(7.0%) | 6(6.8%) | 0(0.0%) | 0(0.0%) |  |  |  |  |
|  | G |  | 129(75.0%) | 134(76.1%) | 105(87.5%) | 325(92.3%) |  |  |  |  |
| **rs59901247** | AA |  | 75(87.2%) | 76(86.4%) | 55(91.7%) | 164(92.1%) |  | 0.176^a^ | 0.115 | 0.159 |
|  | AC |  | 10(11.6%)) | 12(13.6%) | 5(8.3%) | 12(6.7%) |  |  |  |  |
|  | CC |  | 1(1.2%) | 0(0.0%) | 0(0.0%) | 2(1.1%) |  |  |  |  |
|  | A |  | 160(93.0%) | 164(93.2%) | 115(95.8%) | 340(95.5%) |  |  |  |  |
| **rs1868092** | AA |  | 38(44.7%) | 38(43.2%) | 34(56.7%) | 127(72.2%) |  | **5.19E-8** | **4.39E-7** | **2.14E-8** |
|  | AG |  | 36(42.4%) | 40(45.5%) | 22(36.7%) | 45(25.6%) |  |  |  |  |
|  | GG |  | 11(12.9%) | 10(11.4%) | 4(6.7%) | 4(2.3%) |  |  |  |  |
|  | A |  | 112(65.9%) | 116(65.9%) | 90(75.0%) | 299(84.9%) |  |  |  |  |

Abbreviations: Additive, additive model; Dominant, dominant model.

*P*-values except the noted ones are calculated from χ^2^ test.

**^a^** *P*-values are calculated from Fisher exact test.

**^b^** Bold type denotes *P*<0.05.

**Table B. in S1 File Association analysis between *HIF2A* tSNPs and levels of high altitude among native female** Tibetans.

| **SNP** | **Genotype** |  | **Level of high altitude** | | | |  | ***P*-value^b^** | | |
| --- | --- | --- | --- | --- | --- | --- | --- | --- | --- | --- |
|  | **/Allele** |  | **Bomi** | **Qamdo** | **Lhasa** | **Amdo** |  | **Additive** | **Dominant** | **Allelic** |
| **rs56721780** | GG |  | 72(70.6%) | 72(68.6%) | 85(45.5%) | 40(44.4%) |  | **7.64E-7^a^** | **1.16E-6** | **7.82E-7** |
|  | CG |  | 26(25.5%) | 32(30.5%) | 85(45.5%) | 41(45.6%) |  |  |  |  |
|  | CC |  | 4(3.9%) | 1(1.0%) | 17(9.1%) | 9(10.0%) |  |  |  |  |
|  | C |  | 34(16.7%) | 34(16.2%) | 119(31.8%) | 59(32.4%) |  |  |  |  |
| **rs6756667** | AA |  | 51(49.5%) | 60(55.0%) | 116(61.4%) | 73(80.2%) |  | **4.24E-7^a^** | **1.60E-5** | **2.78E-7** |
|  | AG |  | 37(35.9%) | 45(41.3%) | 66(34.9%) | 17(18.7%) |  |  |  |  |
|  | GG |  | 15(14.6%) | 4(3.7%) | 7(3.7%) | 1(1.1%) |  |  |  |  |
|  | A |  | 139(67.5%) | 165(75.7%) | 298(78.8%) | 163(89.6%) |  |  |  |  |
| **rs7589621** | GG |  | 60(58.3%) | 67(61.5%) | 127(67.6%) | 75(84.3%) |  | **2.11E-5^a^** | **1.49E-4** | **1.49E-5** |
|  | AG |  | 33(32.0%) | 39(35.8%) | 56(29.8%) | 13(14.6%) |  |  |  |  |
|  | AA |  | 10(9.7%) | 3(2.8%) | 5(2.7%) | 1(1.1%) |  |  |  |  |
|  | G |  | 153(74.3%) | 173(79.4%) | 310(82.4%) | 163(91.6%) |  |  |  |  |
| **rs59901247** | AA |  | 86(83.5%) | 90(82.6%) | 172(91.5%) | 79(86.8%) |  | 0.121^a^ | 0.121 | 0.134 |
|  | AC |  | 17(16.5%) | 19(17.4%) | 16(8.5%) | 12(13.2%) |  |  |  |  |
|  | CC |  | 0(0.0%) | 0(0.0%) | 0(0.0%) | 0(0.0%) |  |  |  |  |
|  | A |  | 189(91.7%) | 199(91.3%) | 360(95.7%) | 170(93.4%) |  |  |  |  |
| **rs1868092** | AA |  | 43(41.7%) | 48(44.4%) | 101(54.4%) | 63(70.0%) |  | **9.91E-7** | **4.32E-5** | **1.23E-6** |
|  | AG |  | 43(41.7%) | 52(48.1%) | 79(42.2%) | 24(26.7%) |  |  |  |  |
|  | GG |  | 17(16.5%) | 8(7.4%) | 7(3.7%) | 3(3.3%) |  |  |  |  |
|  | A |  | 129(62.6%) | 148(68.5%) | 281(75.1%) | 150(83.3%) |  |  |  |  |

Abbreviations: Additive, additive model; Dominant, dominant model.

*P*-values except the noted ones are calculated from χ^2^ test.

**^a^** *P*-values are calculated from Fisher exact test.

**^b^** Bold type denotes *P*<0.05.
